# Growth patterns in coeliac disease – a longitudinal study of children aged 0–6 years in Sweden

**DOI:** 10.1186/s12887-026-06903-6

**Published:** 2026-05-09

**Authors:** Lisa Andersson Fjelstad, Annelie Lindholm, Julia S. Malmborg, Awais Ashfaq, Lars Gelander, Anton Holmgren

**Affiliations:** 1 School of Health and Welfare, Halmstad University, Halmstad, Sweden; 2 Department of Research and Development, Region Halland, Halmstad, Sweden; 3 School of ITE, Halmstad University, Halmstad, Sweden; 4 Department of Paediatrics, Institute of Clinical Sciences, Sahlgrenska Academy, University of Gothenburg, Gothenburg, Sweden; 5 Department of Paediatrics, Halland Hospital, Halmstad, Sweden

**Keywords:** Catch-up growth, Coeliac disease, Growth deviation, Healthcare, Paediatric care

## Abstract

**Background:**

Associations between growth deviations and coeliac disease (CD) in children have been documented, but longitudinal evidence—particularly concerning when such deviations first emerge—is limited. The aim of this study was to investigate the occurrence and timing of growth deviations in relation to CD diagnosis, and to assess whether these patterns differed between boys and girls in a Swedish preschool population.

**Methods:**

This retrospective longitudinal study was conducted as part of the project *Evidence based knowledge about deviant growth in children 0–6 years*. A total of 185 children with CD were identified through the Regional Healthcare Information Platform. Background characteristics and growth deviations relative to the time of CD diagnosis were analysed using descriptive statistics and tests, including chi square, Mann–Whitney U, one way ANOVA, Kruskal–Wallis, paired t tests, and Wilcoxon signed rank tests.

**Results:**

Of the 185 participating children, 64 (34.6%) were boys and 121 (65.4%) were girls. Growth deviations were most pronounced at the time of diagnosis, with mean weight SDS of − 0.54 (± 1.2) and mean height SDS of − 0.48 (± 1.1). No significant sex differences were observed. Children diagnosed between 36 and 48 months exhibited significantly greater negative height SDS deviations at one year and six months prior to diagnosis compared with younger age groups. Significant improvements in both weight SDS and height SDS were observed during the two years following diagnosis and initiation of treatment.

**Conclusion:**

Growth deviations varied according to age at CD diagnosis, with the most pronounced deviations occurring in proximity to the diagnostic timepoint. Growth improved significantly during the two years following diagnosis. These findings suggest that subtle growth deviations may be detectable years before diagnosis, supporting the clinical value of careful growth monitoring in paediatric care. Further research on the implementation of growth based screening parameters in routine practice is warranted.

**Supplementary Information:**

The online version contains supplementary material available at 10.1186/s12887-026-06903-6.

## Background

Coeliac disease (CD) is a genetically predisposed autoimmune disorder characterized by an immunological reaction to the gluten proteins found in wheat, barley, and rye [1]. The condition is typically diagnosed through serological testing, most commonly by detecting elevated transglutaminase antibodies [1]. CD has an estimated global seroprevalence of 1.4% and a biopsy‑confirmed prevalence of 0.7% [2, 3]. It is more common in females (0.6%) than in males (0.5%) and is diagnosed more frequently in children (0.8%) than in adults (0.5%) [2, 3].

Symptoms of CD in children vary widely [1], and deviations from expected growth patterns have been documented in several studies [4–6]. However, only a limited number of investigations have examined when such growth deviations emerge in relation to the timing of diagnosis [7]. Sex‑related differences have also been reported, with some studies suggesting that growth deviations may appear earlier or be more pronounced in girls than in boys [8, 9]. Following diagnosis, adherence to a gluten‑free diet often leads to improvements in growth, commonly referred to as catch‑up growth [10]. Early diagnosis is crucial because prolonged untreated CD can negatively affect growth and may increase the risk of complications such as adenocarcinoma and lymphoma [10–12]. Because growth deviations may represent an early clinical sign of CD in children, identifying such patterns is important for paediatric healthcare [7]. It may therefore be of value with additional research, performed in a current Swedish context, to see if Swedish children also present growth deviations in paediatric care, due to CD.

The objective of this study was to determine whether measurable growth deviations occur in children with CD before diagnosis, to examine how these deviations vary by age at diagnosis and sex, and to assess whether such patterns could support earlier clinical detection.

## Methods

### Study design and population

An ongoing project in the county of Halland in south‑west Sweden, *Evidence‑based knowledge about deviant growth in children 0–6 years (EKAT‑06)*, investigates how growth deviations in young children relate to factors such as specific diseases, socioeconomic conditions, and healthcare utilisation. The project is based on the Regional Healthcare Information Platform (RHIP), a database containing medical record information from approximately 56,000 children registered in Halland between 1 January 2009 and 31 December 2022 [13, 14].

The present study used a retrospective longitudinal design and was conducted as a subproject within EKAT‑06. It included all children aged 0–6 years diagnosed with CD during the study period, forming a defined preschool cohort connected to the main project. CD diagnoses were identified through ICD‑10 coding (K90.0), in RHIP, which in Swedish paediatric practice corresponds to ESPGHAN‑aligned diagnostic procedures based on serology and/or biopsy when clinically indicated.

**Fig. 1:**
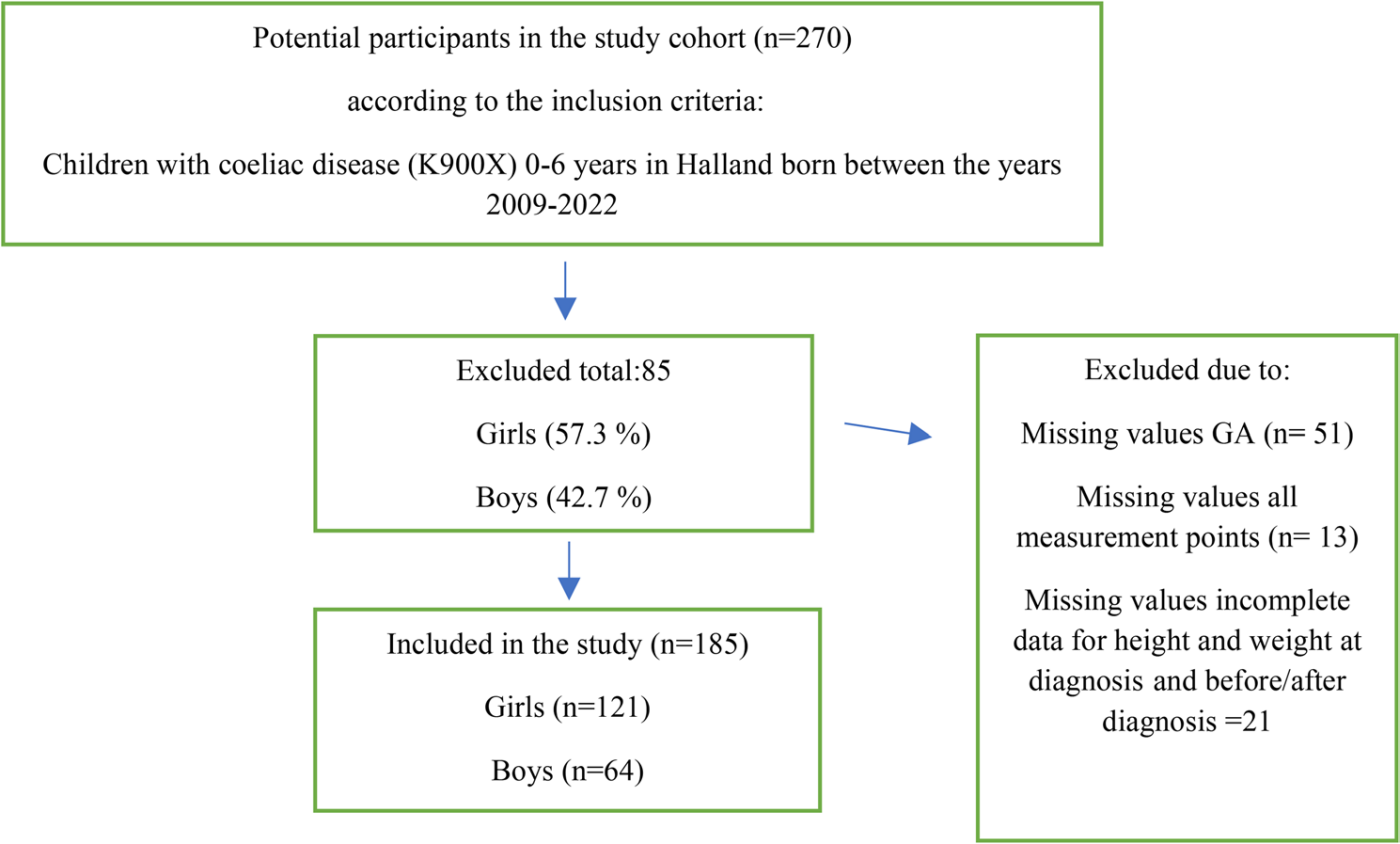
Potential participants and selected participants in the study according to inclusion and exclusion criteria

A total of 270 potential participants with CD were identified. To minimise bias, children were excluded if gestational age data were missing, if anthropometric data were incomplete (e.g., due to moving to or from the region), or if no measurements were available at any of the required timepoints. Gestational age, (GA) was required for accurate interpretation of birth weight and birth length. Without GA, classification relative to population growth standards would not have been possible, compromising comparability of baseline characteristics. After applying exclusion criteria, 185 children remained in the analytical cohort (Fig. 1).

### Data collection

Anthropometric measurements (weight and height/length) were obtained from the RHIP database based on standardised Child Health Care (CHC) visits. Measurements were conducted by trained Child Health Services (CHS) nurses using calibrated equipment following national guidelines [15, 16]. For children under 2 years of age, length was measured in a supine position using stadiometers, and weight was measured unclothed on an infant scale (up to 15 kg). For children older than 2 years, standing height was measured with a stadiometer, and body weight was measured in underpants on an electronic or mechanical step scale.

### Outcome measures

The primary outcomes were weight, height, and deviations in these measures expressed as weight‑SDS and height‑SDS. SDS values were calculated using Swedish national reference standards for age and sex [17, 18]. Growth data were analysed at seven clinically relevant timepoints relative to the CD diagnosis: 2 years before, 1 year before, 6 months before, at diagnosis, 6 months after, 1 year after, and 2 years after. Measurements closest to each timepoint (± 4 months) were accepted; if no measurement fell within this window, the value was classified as missing. Complete case analysis was used because missing data could reflect the absence of entire time anchored measurement points rather than isolated values.

Age‑at‑diagnosis groups were categorised into the following intervals: 0–12 months, 12–24 months, 24–36 months, 36–48 months, 48–60 months, and 60–72 months. Analyses were also stratified by sex.

Background characteristics included age at diagnosis, sex distribution, mean (± SD) and median (IQR). Birth weight and length were categorised into: < 2.5 kg, 2.5–4.5 kg (within ± 2 SD), and > 4.5 kg for birth weight; and < 47 cm, 47–54.5 cm (within ± 2 SD), and > 54.5 cm for birth length. Weight‑SDS and height‑SDS at birth were categorised as < − 1 SDS, within ± 1 SDS, and > + 1 SDS. Gestational age categories were defined as preterm (< 37 weeks / < 259 days), term (37–41.9 weeks / 259–293 days), and post‑term (≥ 42 weeks / ≥ 294 days) [19].

### Statistical methods

Statistical analyses were performed using IBM SPSS Statistics (version 29.0.1.0; IBM Corp., Armonk, NY, USA). Descriptive statistics included frequencies, means (± SD), and medians (IQR). Normality was assessed using skewness, kurtosis, Kolmogorov–Smirnov, and Shapiro–Wilk tests. Missing values were reported as “missing.”

To compare continuous variables between boys and girls, independent t‑tests or Mann–Whitney U‑tests were used depending on distribution. One‑way ANOVA and Kruskal–Wallis tests were applied for comparisons across more than two groups, with Tukey’s HSD or Dunn’s post‑hoc tests used when applicable. Differences in proportions were assessed using non-parametric binominal test and chi‑square tests. Longitudinal changes between paired timepoints were analysed using paired t‑tests or Wilcoxon matched‑pair signed‑rank tests for non‑normally distributed data. A p‑value < 0.05 was considered statistically significant.

### Ethical considerations

The EKAT‑06 project was approved by the Swedish Ethical Review Authority (No. 2022‑06743‑01). As the study used previously collected registry data, individual informed consent was not required under the conditions of the ethics approval [20]. Information about the possibility to opt out was available on the Region Halland website, allowing parents to withdraw their child’s data if desired [21].

## Results

A total of 185 children were included in the study (64 boys and 121 girls). Most participants (94.6%) were born at term and had birth weight (79.5%) and birth length (75.7%) values within normal population reference ranges used in clinical assessment (birth weight 2.5–4.5 kg; birth length 47–54.5 cm). Weight‑SDS and height‑SDS at birth indicated some early growth deviations, with 21.1% of the children classified as < − 1 weight‑SDS and 26.5% as < − 1 height‑SDS. A statistically significant sex difference was found in baseline characteristics for weight‑SDS at birth and mean birth length (Table 1).

**Table 1:** Baseline characteristics of the study population grouped according to sex and presented as percentage, mean (± SD) or median (IQR)

	Total, *n* (%) or mean ± SD	mdn (IQR)	Boys, *n* (%) or mean ± SD	mdn (IQR)	Girls, *n* (%) or mean ± SD	mdn (IQR)	*p*-value
Child characteristics							
Number of children	185 (100)		64 (34.6)		121 (65.4)		**< 0.001**
Gestational age (GA)							
Preterm^a^	7 (3.8)		4 (6.3)		3 (2.5)		
Term^b^	175 (94.6)		58 (90.6)		117 (96.7)		
Post term^c^	3 (1.6)		2 (3.1)		1 (0.8)		0.237^j^
Weight at birth							
Birth weight grouping							
Below average^d^	5 (2.7)		2 (3.1)		3 (2.5)		
Average^e^	147 (79.5)		55 (85.9)		92 (76.0)		1.000^k^
Above average^f^	2 (1.1)		0		2 (1.7)		
Birth weight (kg)	3.45 (± 0.51)	3.47 (0.74)	3.53(± 0.49)	3.58(0.6)	3.41 (± 0.52)	3.40 (0.77)	0.055
Missing values, *n* = 31							
Weight-SDS at birth							
< -1 weight-SDS	39 (21.1)		8 (12.5)		31 (25.6)		
Within 1 weight-SDS	99 (53.5)		43 (67.2)		56 (46.3)		
> 1 weight-SDS	16 (8.6)		6 (9.4)		10 (8.3)		**0.031**
Missing values, *n* = 31							
Length at birth							
Birth length grouping							
Below average^g^ Average^h^	7 (3.8) 140 (75.7)		0 53 (82.8)		7 (5.8) 87 (71.9)		
Above average^i^	1 (0.5)		1 (1.6)		0		**0.048** ^j^
Birth length (cm)	50.1 (± 2.1)	50.0 (3.0)	50.7 (± 1.8)	50.5 (2.3)	49.7 (± 2.2)	50.0 (3.0)	**0.017**
Missing values,* n* = 37							
Height-SDS at birth							
< -1 height-SDS	49 (26.5)		13 (20.3)		36 (29.8)		
Within 1 height-SDS	82 (44.3)		36 (56.3)		46 (38.0)		
> 1 height-SDS	17 (9.2)		5 (7.8)		12 (9.9)		0.128
Missing values, *n* = 37							

Differences between boys and girls were analysed by nonparametric binomial test, by Chi-2 test for categorical variables, and Mann Whitney U-test for birth weight and length (not normally distributed). 0.05 level of significance, significant results in bold

*Abbreviations*: *Weight-SDS*  Weight standard deviation score, *Height-SDS*  Height standard deviation score, *IQR* Interquartile range, *mdn* median

^a^Preterm < 37 weeks = < 259days. ^b^Term 37-41.9 weeks = 259–293 days ^c^Post term ≥ 42 weeks = ≥ 294 days Birth weight grouping: ^d^Below average (< 2.5 kg) ^e^Average (2.5–4.5 kg) ^f^Above average (> 4.5 kg). Birth length grouping: ^g^Below average (< 47 cm), ^h^Average (47–54.5 cm), ^i^Above average (> 54.5 cm). ^j^Postterm was combined with term for gestational age and above average was combined with average for birth length grouping when performing Chi 2 test, Fisher´s exact was used for p-value due to counts < 5 in groups in these variables. ^k^For birth weight grouping, above average was combined with average when performing Chi-2 test and Fisher´s exact was used for p-value due to count < 5

Age at diagnosis also differed significantly between boys and girls. Boys were diagnosed earlier (median 3.3 years) than girls (median 3.8 years) (Table 2).

**Table 2:** Age at diagnosis for girls and boys presented as (%), mean (± SD) and median (IQR)

	Total, *n* (% ) or mean ± SD *n* = 185	mdn (IQR) *n* = 185	Boys, *n* (%) or mean ± SD *n* = 64	Boys mdn (IQR) *n* = 64	Girls, *n* (%) or mean ± SD *n* = 121	Girls mdn (IQR) *n* = 121	*p*-value
Age at diagnosis							
0–12 months	7 (3.8)		5 (7.8)		2 (1.7)		
12,01–24 months	30 (16.2)		13 (20.3)		17 (14.0)		
24,01–36 months	30 (16.2)		12 (18.8)		18 (14.9)		
36,01–48 months	39 (21.1)		11 (17.2)		28 (23.1)		
48,01–60 months	33 (17.8)		14 (21.9)		19 (15.7)		
60,01–72 months	46 (24.9)		9 (14.1)		37 (30.6)		
Age at diagnosis (years)	3.6 (± 1.5)	3.8 (2.6)	3.2 (± 1.6)	3.3 (2.5)	3.8 (± 1.4)	3.8 (2.4)	**0.019**

Differences between the median age at diagnosis in years between boys and girls were analysed with Mann-Whitney U-test. 0.05 level of significance, significant results in bold

*Abbreviations*: *SD* standard deviation, *IQR* Interquartile range, *mdn* median

Growth deviations in weight‑SDS and height‑SDS were evaluated at seven measurement points relative to the CD diagnosis. Mean and median values at these timepoints showed that growth deviations were greatest shortly before diagnosis and at the time of diagnosis. No statistically significant sex differences were observed for weight‑SDS or height‑SDS at any of the measurement points. Growth deviations in both weight‑SDS and height‑SDS had normalised by two years after diagnosis for both sexes, likely reflecting the effect of treatment with a gluten‑free diet (Table 3 and 4).

**Table 3:** Growth deviations presented as weight-SDS and height-SDS in the study population presented as mean (± SD), or median (IQR)

	Total mean (± SD) *n* = 185	Total mdn (IQR) *n* = 185	CI (95%) *n* = 185	Boys, mean (± SD) *n* = 64	Boys mdn (IQR) *n* = 64	Girls, mean (± SD) *n* = 121	Girls mdn (IQR) *n* = 121 p-value
Time prior / after diagnosis							
2 years prior							
weight-SDS	-0.38 (± 1.2)	-0.49 (1.5)	-0.60-(-0.16)	-0.22 (± 1.3)	-0.14 (2.0)	-0.46 (± 1.1)	-0.50 (1.0) 0.302^b^
Missing, *n* = 69							
height-SDS	-0.11 (± 1.1)	-0.09 (1.4)	-0.32-(0.09)	-0.19 (± 1.1)	-0.29 (1.8)	-0.07 (± 1.1)	-0.02 (1.1) 0.603^b^
Missing, *n* = 71							
1 year prior							
weight-SDS	-0.40 (± 1.2)	-0.38 (1.4)	-0.59-(-0.20)	-0.37 (± 1.2)	-0.25 (1.9)	-0.41 (± 1.2)	-0.40 (1.3) 0.932^a^
Missing, *n* = 34							
height-SDS	-0.19 (± 1.1)	-0.18 (1.5)	-0.37-(-0.00)	-0.22 (± 1.1)	-0.19 (1.9)	-0.17 (± 1.1)	-0.18 (1.3) 0.704^a^
Missing, *n* = 43							
6 months prior							
weight-SDS	-0.55 (± 1.2)	-0.44 (1.6)	-0.74-(-0.36)	-0.49 (± 1.3)	-0.56 (1.9)	-0.58 (± 1.1)	-0.43 (1.5) 0.565^a^
Missing, *n* = 31							
height-SDS	-0.40 (± 1.1)	-0.39 (1.5)	-0.58-(-0.22)	-0.41 (± 1.1)	-0.60 (1.7)	-0.39 (± 1.2)	-0.36 (1.3) 0.910^b^
Missing, *n* = 34							
At diagnosis							
weight-SDS	-0.54 (± 1.1)	-0.48 (1.5)	-0.71-(-0.38)	-0.53 (± 1.2)	-0.50 (1.8)	-0.55 (± 1.1)	-0.46 (1.4) 0.927^a^
Missing, *n* = 1							
height-SDS	-0.48 (± 1.1)	-0.50 (1.5)	-0.65-(-0.32)	-0.54 (± 1.1)	-0.53 (1.7)	-0.46 (± 1.1)	-0.46 (1.5) 0.649^b^
Missing, *n* = 4							
6 months after							
weight-SDS	-0.32 (± 1.2)	-0.28 (1.5)	-0.50-(-0.14)	-0.45 (± 1.2)	-0.50 (1.8)	-0.25 (± 1.1)	-0.24 (1.4) 0.324^a^
Missing, *n* = 26							
height-SDS	-0.46 (± 1.1)	-0.47 (1.4)	-0.63-(-0.29)	-0.58 (± 1.0)	-0.64 (1.4)	-0.40 (± 1.1)	-0.39 (1.4) 0.180^a^
Missing, *n* = 29							
1 year after							
weight-SDS	-0.23 (± 1.2)	-0.26 (1.6)	-0.43-(-0.03)	-0.16 (± 1.3)	-0.28 (1.8)	-0.27 (± 1.2)	-0.26 (1.5) 0.598^b^
Missing, *n* = 32							
height-SDS	-0.36 (± 1.1)	-0.24 (1.3)	-0.54-(-0.17)	-0.36 (± 1.0)	-0.18 (1.3)	-0.36 (± 1.2)	-0.30 (1.4) 0.852^a^
Missing, *n* = 36							
2 years after							
weight-SDS	-0.15 (± 1.3)	-0.14 (1.6)	-0.38-(0.09)	-0.10 (± 1.4)	-0.28 (1.8)	-0.17 (± 1.2)	-0.08 (1.6) 0.806^b^
Missing, *n* = 70							
height-SDS	-0.27 (± 1.2)	-0.06 (1.4)	-0.49-(-0.05)	-0.25 (± 1.1)	-0.06 (1.4)	-0.28 (± 1.2)	-0.06 (1.4) 0.937^a^
Missing, *n* = 72							

Differences between boys and girls at measurement points were analysed by independent samples t-test and Mann Whitney U-test (for non-normal distributed data).

Abbreviations: *Weight-SDS*= Weight standard deviation score *Height-SDS* = Height standard deviation score. *SD*= standard deviation *IQR*= Interquartile range *mdn* =median

^a^Mann whitney U test, ^b^Independent samples t-test

**Table 4:** Differences in time between measurement points for growth deviations, presented as *p-*value

Difference between time points	*p*-value
weight-SDS	
2 y before and 1 y before diagnosis	**0.015**^**a**^
height-SDS	
2 y before and 1 y before diagnosis	**<0.001**^**a**^
weight-SDS	
1 y and 6 m before	**0.019**^**a**^
height-SDS	
1 y and 6 m before	**0.003**^**a**^
weight-SDS	
6 m before and at diagnosis	0.676^a^
height-SDS	
6 m before and at diagnosis	**< 0.001**^**b**^
weight-SDS	
At diagnosis and 6 m after	**< 0.001**^**a**^
height-SDS	
At diagnosis and 6 m after	**< 0.001**^**a**^
weight-SDS	
6 m after and 1 y after	**0.129**^**b**^
height-SDS	
6 m after and 1 y after	0.113^a^
weight-SDS	
1 y ad 2 y after	0.075^b^
height-SDS	
1 y ad 2 y after	**0.001**^**a**^
weight-SDS	
At diagnosis and 2 y after	**< 0.001**^**a**^
height-SDS	
At diagnosis and 2 y after	**< 0.001**^**a**^

Differences between two different measurement points were analysed by Paired t-test and Wilcoxon matched-pair signed rank test (for data not normally distributed).  0.05 level of significance, significant results in bold

Abbreviations: *Weight-SDS =* Weight standard deviation score. *Height-SDS* = Height standard deviation score. *SD*= standard deviation.

^a﻿^Wilcoxon signed rank test, ^b^Paired t-test

When comparing age‑at‑diagnosis groups (0–12 months, 12–24 months, 24–36 months, 36–48 months, 48–60 months, and 60–72 months), significant differences between groups were observed for height‑SDS at one year prior to diagnosis and six months prior to diagnosis (Table 5). Post‑hoc analyses showed that children diagnosed at 36–48 months had significantly greater negative height‑SDS deviations six months before diagnosis than those diagnosed at 12–24 months. At one year prior to diagnosis, Dunn’s post‑hoc test showed that the 36–48-month group had significantly larger negative height‑SDS deviations compared with both the 12–24 month and 24–36-month groups. Additionally, children diagnosed at 60–72 months showed significantly more reduced height‑SDS values than those diagnosed at 12–24 months.

**Table 5:** Growth deviations expressed as weight-SDS and height-SDS in the study population at different time stamps presented as mean (± SD), or median (IQR) for the different groups defined by age in months at diagnosis

Time prior and after diagnosis	0–12 m mean (± SD) /mdn(IQR) *n* = 7	12.01–24 m mean (± SD) /mdn (IQR) *n* = 30	24.01–36 m mean (± SD) /mdn (IQR) *n* = 30	36.01–48 m mean (± SD) /mdn (IQR) *n* = 39	48.01–60 m mean (± SD) /mdn (IQR) *n* = 33		60.01–72 m mean (± SD) /mdn (IQR) *n* = 46
2 years prior							
weight-SDS	-	-	-0.12 (± 1.5) / -0.03 (2.1)	-0.63 (± 1.1)/ -0.63 (1.1)	-0.38 (± 1.1)/ -0.12 (2.0)		-0.30 (± 0.8)/ -0.30 (1.0)
* p-value*							*0.339* ^*a c*^
height-SDS	-	-	0.28 (± 1.2)/ 0.48(1.5)	-0.48 (± 1.2)/ -0.45 (1.3)	-0.07 (± 1.1)/ -0.07 (2.1)		-0.08 (± 0.6)/ -0.05 (0.6)
* p-value*							*0.067* ^*a d*^
1 year prior							
weight-SDS	-	-0.29 (± 1.3)/ -0.11 (1.5)	-0.43 (± 1.4)/ -0.48 (1.8)	-0.82 (± 1.0)/ -0.75 (1.0)	-0.09 (± 1.2)/ -0.10 (1.6)		-0.29 (± 1.2)/ -0.49 (1.3)
* p-value*							*0.108* ^*b*^
height-SDS	-	0.06 (± 1.0)/ 0.19 (1.2)	0.15 (± 1.1)/ 0.03(1.3)	-0.68 (± 1.3)/ -0.71 (2.1)	-0.03 (± 1.2)/ 0.07 (2.0)		-0.34 (± 0.8)/ -0.37 (1.4)
* p-value*							***0.029*** ^*b d*^
6 months prior							
weight-SDS	-1.0 (± 2.0)/ -0.68 (-)	-0.44 (± 1.2)/ -0.25 (2.0)	-0.96 (± 1.3)/ -0.84 (2.2)	-0.79 (± 0.9)/ -0.66 (1.5)	-0.19 (± 1.1)/ -0.24 (1.4)		-0.36 (± 1.2)/ -0.42 (1.3)
* p-value*							*0.225* ^*d*^
height-SDS	-0.71(± 1.5)/ -1.4 (-)	-0.07 (± 1.0)/ -0.02 (1.2)	-0.26 (± 1.1)/ -0.28 (1.5)	-0.96 (± 1.2)/ -0.84 (1.2)	-0.15 (± 1.3)/ 0.15 (1.7)		-0.40 (± 0.9)/ -0.62 (1.4)
* p-value*							***0.029*** ^*c*^
* MD*							0.888
At diagnosis							
weight-SDS	0.23 (± 1.7)/ 0.71 (2.7)	-0.80 (± 1.2)/ -0.80 (1.5)	-0.66 (± 1.3)/ -0.38 (2.0)	-0.71 (± 0.9)/ -0.58 (1.4)	-0.36 (± 1.1)/ -0.32 (1.6)		-0.41 (± 1.1)/ -0.46 (1.6)
* p-value*							*0.431* ^*d*^
height-SDS	0.48 (± 1.7)/ 1.25 (1.8)	-0.56 (± 1.1)/ -0.43 (1.3)	-0.37 (± 1.9)/ -0.31 (1.4)	-0.89 (± 1.2)/ -0.93 (1.5)	-0.39 (± 1.2)/ -0.27 (1.6)		-0.39 (± 0.9)/ -0.50 (1.4)
* p-value*							*0.050* ^*c*^
6 months after							
weight-SDS	0.10 (± 2.0)/ 0.34 (2.9)	-0.24 (± 1.1)/ -0.27 (1.3)	-0.48 (± 1.3)/ -0.21 (2.1)	-0.62 (± 0.9)/ -0.57 (1.3)	-0.27 (± 1.2)/ -0.28 (2.0)		-0.10(± 1.2)/ -0.20 (1.5)
* p-value*							*0.498* ^*d*^
height-SDS	0.25 (± 1.4)/ 0.66 (1.2)	-0.50 (± 1.1)/ -0.50 (1.0)	-0.41 (± 1.1)/ -0.44 (1.6)	-0.85 (± 1.1)/ -0.80 (1.3)	-0.44 (± 1.2)/ -0.43 (1.6)		-0.26 (± 0.8)/ -0.28 (1.1)
* p-value*							*0.057* ^*d*^
1 year after							
weight-SDS	0.14 (± 1.8)/ 0.43 (3.2)	-0.00 (± 1.1)/ -0.12 (1.1)	-0.16 (± 1.5)/ 0.04(2.3)	-0.61 (± 0.9)/ -0.51 (1.1)	-0.24 (± 1.3)/ -0.37 (1.6)		-0.15 (± 1.3)/ -0.22 (1.7)
* p-value*							*0.426* ^*c*^
height-SDS	0.19 (± 1.4)/ 0.62 (1.8)	-0.33 (± 1.0)/ -0.36 (0.9)	-0.19 (± 1.1)/ -0.07 (1.6)	-0.82 (± 1.3)/ -0.79 (1.6)	-0.32 (± 1.2)/ -0.10 (1.4)		-0.21 (± 0.9)/ 0.01 (1.2)
* p-value*							*0.095* ^*d*^
2 years after							
weight-SDS	0.19 (± 1.0)/ 0.57 (1.6)	0.11 (± 1.0)/ -0.10 (1.3)	-0.06 (± 1.7)/ 0.10(2.4)	-0.45 (± 1.0)/ -0.46 (1.4)	-0.28 (± 1.3)/ -0.49 (1.8)		-0.08 (± 1.5) -0.39 (1.6)
* p-value*							*0.563* ^*d*^
height-SDS	0.20 (± 0.9)/ 0.53 (1.2)	-0.14 (± 1.3)/ -0.01 (1.1)	0.04 (± 1.2)/ 0.26(1.4)	-0.68 (± 1.4)/ -0.54 (1.9)	-0.39 (± 1.2)/ -0.51 (1.5)		-0.26 (± 0.9)/ -0.24 (1.5)
* p-value*							*0.197* ^*d*^

Differences between the groups were analysed by One-way ANOVA and Kruskal-Wallis-test (for data not normally distributed). 0.05 level of significance, significant results in bold

Abbreviations: *Weight-SDS*= Weight standard deviation score. *Height-SDS* = height standard deviation score. *SD*= standard deviation *IQR*= Interquartile range *mdn* =median *MD*=Mean difference

^a^Combined the groups 0–12 months and 12.01-24 months with the group 24.01-36 months, when performing Kruskal-Wallis test, due to lack of participants in these groups at this measurement point. ^b^Combined the group 0–12 months with the group 12.01-24 months when performing Kruskal-Wallis test, due to lack of participants in this group at this measurement point. ^c^One way ANOVA. ^d^Kruskal Wallis test

Scatterplots of weight‑SDS and height‑SDS one year prior to diagnosis (Supplemental Fig. S1 and S2), further illustrated these patterns. Weight‑SDS deviations appeared more pronounced than height‑SDS deviations at this timepoint, while height‑SDS deviations tended to appear later in the disease course.

## Discussion

Children with CD in this study exhibited modest growth deviations—approximately − 0.5 SDS for both weight and height—across all measured timepoints. These findings are consistent with previous research involving younger children [7], although the earlier study included children aged 0–2.5 years. While growth deviations were detectable as early as two years before diagnosis, their relatively small magnitude may limit their usefulness for early clinical detection. No significant sex‑based differences in growth were observed, consistent with findings from one study assessing growth failure [8] but contrasting with other studies reporting earlier or more pronounced deviations in girls [8, 9]. These inconsistencies highlight ongoing uncertainty in the literature and underscore the need for further research examining sex‑specific growth patterns in CD.

A statistically significant sex difference was found at birth for weight‑SDS and mean birth length (Table 1), which is in line with established population growth references [17, 18]. In this cohort, approximately two‑thirds of the diagnosed children were girls. This mirrors several clinical studies reporting a higher proportion of diagnosed girls than boys. Importantly, these proportions differ from global seroprevalence estimates [2, 3], which reflect broader epidemiological estimates rather than clinical diagnostic cohorts. Similar distributions have been described in multiple studies [5, 8, 9, 22], including one showing 60% girls and 40% boys among children diagnosed with CD [9]. Although girls with Turner syndrome have an increased risk of CD, the prevalence of Turner syndrome is too low to significantly influence sex distribution [23]. Some studies suggest that symptom presentation in girls may be more distinct and therefore more likely to prompt diagnostic testing [24, 25], while others propose an increased genetic susceptibility in girls [24, 26, 27].

Despite the higher number of diagnosed girls, boys in this study were diagnosed at a significantly younger age. Similar findings have been reported elsewhere [27]. The reason underlying this discrepancy remains unclear, particularly given the expectation that girls—who may have a higher predisposition or clearer symptom profiles—would be diagnosed earlier. This suggests a knowledge gap regarding sex‑related differences in diagnostic trajectories [24–27].

The results indicate that children with potential CD are closely monitored within the Child Health Services (CHS) system, as growth deviations were greatest at the time of diagnosis and decreased thereafter. Identifying CD at an earlier stage based solely on growth measures is challenging, given that the deviations observed were modest. A deviation of − 0.5 weight‑SDS corresponds to approximately 0.5 kg at ages 2–4 years and 1–1.25 kg at ages 5–6 years, while height‑SDS deviations correspond to roughly 1 cm at ages 2–3, 2 cm at ages 4–5, and 2.5 cm at age 6 [17]. Scatterplot analyses (Figs. S1 and S2) further showed that children diagnosed at younger ages often exhibited more pronounced weight‑SDS deviations one year before diagnosis, possibly reflecting more severe forms of CD, as disease severity may vary across individuals [4–9, 28].

Significant differences by age of diagnosis were noted for height‑SDS at the one‑year and six‑month timepoints prior to diagnosis. Children diagnosed between 36 and 48 months showed the largest negative deviations, indicating that age at diagnosis may influence the expression of growth impairment. Height tends to respond more slowly than weight to disease processes, both before and after diagnosis, which may partially explain the observed group differences. Interpreting growth deviations in children under two years is particularly complex, as growth velocity normally slows during this developmental period, and early symptoms of CD may be subtle or non‑specific [29]. The observation that growth deviations may begin up to two years before diagnosis emphasizes the need for heightened clinical vigilance. Even small but persistent deviations should prompt consideration of CD testing, as earlier diagnosis has important implications for child health and well‑being.

The study also demonstrated clear evidence of catch‑up growth following diagnosis [10]. Significant improvements in both weight‑SDS and height‑SDS were observed within two years after initiation of a gluten‑free diet. This aligns with established patterns of recovery in children with CD [10] and with other studies reporting growth normalization after treatment [4, 6, 10]. The extent of growth recovery may depend on factors such as age at diagnosis, adherence to a gluten‑free diet, socioeconomic circumstances, and follow‑up engagement [4, 6, 10].

The study benefits from a large regional cohort and standardized growth monitoring practices. Halland is the seventh most child-rich county of a total of 21 regions in Sweden [30], and the register contains data from cohorts from birth to 6 years of age, which establish representability of the data on population level. The high coverage rate of CHS visits (95% of Swedish children in 2021) ensures robust population‑level data [15, 31].

Nonetheless, several methodological considerations must be addressed. Exclusion of 31.5% of potential participants introduces a risk of selection bias. Missing values were more common at the earliest and latest measurement intervals (2 years before and 2 years after diagnosis), partly due to visit schedules and age‑related variation in measurement frequency. Since the appointments at CHS are more frequent in the early years (up to 2.5 years), some of the missing values for children diagnosed later can be explained by less frequent ordinary visits after this age [15]. Children with regular healthcare attendance are more likely to have complete growth data, meaning excluded children may differ systematically from those included, potentially limiting generalisability. Furthermore, as this was an observational study, unmeasured confounders—such as socioeconomic status, parental height, breastfeeding history, and parental education—may have influenced growth outcomes [32, 33].

Despite these limitations, the findings have important clinical implications. Although growth deviations were modest, they were detectable up to two years before diagnosis, consistent with earlier research showing that slight but persistent reductions in SDS may indicate undiagnosed [7, 22, 28]. Importantly, many children in this study maintained normal or even increasing growth trajectories before diagnosis, underscoring that CD can occur without obvious growth faltering. Clinicians should therefore remain alert to the possibility of CD even when growth appears normal, especially when other subtle symptoms or risk factors are present.

## Conclusions

In summary, small but detectable growth deviations were present up to two years before CD diagnosis. The findings of growth deviations regarding weight and height were most prominent at diagnosis. These deviations were modest and similar between sexes. Growth improved significantly within two years after diagnosis. Greater clinical attention to subtle, persistent deviations may support earlier CD detection.

## Supplementary Information

Supplementary Material 1.

Supplementary Material 2.

## Data Availability

The datasets generated and/or analysed during the current study are not publicly available due to confidentiality and restrictions to the availability of these data, regulated in the General Data Protection Regulation (GDPR). The data can be available after reasonable request and application from the corresponding author.

## Abbreviations

CD: Coeliac disease
CHC: Child Health Care
CHS: Child Health Services
CI: Confidence Interval
EKAT: 06–Evidence based knowledge about deviant growth in children 0–6 years
GA: Gestational age
Height: SDS–Standard deviation score for height
IQR: Interquartile range
Mdn: Median
SD: Standard deviation
SDS: Standard deviation score
Weight: SDS–Standard deviation score for weight
RHIP: Regional Healthcare Information Platform
