# Supplementary material for: Growth patterns in coeliac disease – a longitudinal study of children aged 0–6 years in Sweden
Source: BMC Pediatr. 2026 May 9;26:427. doi: 10.1186/s12887-026-06903-6 (PMC13159342; doi:10.1186/s12887-026-06903-6)
Supplement: Supplementary file 1 — Supplementary Material 1. [file 12887_2026_6903_MOESM1_ESM.docx]

**Supplemental Figure 1**


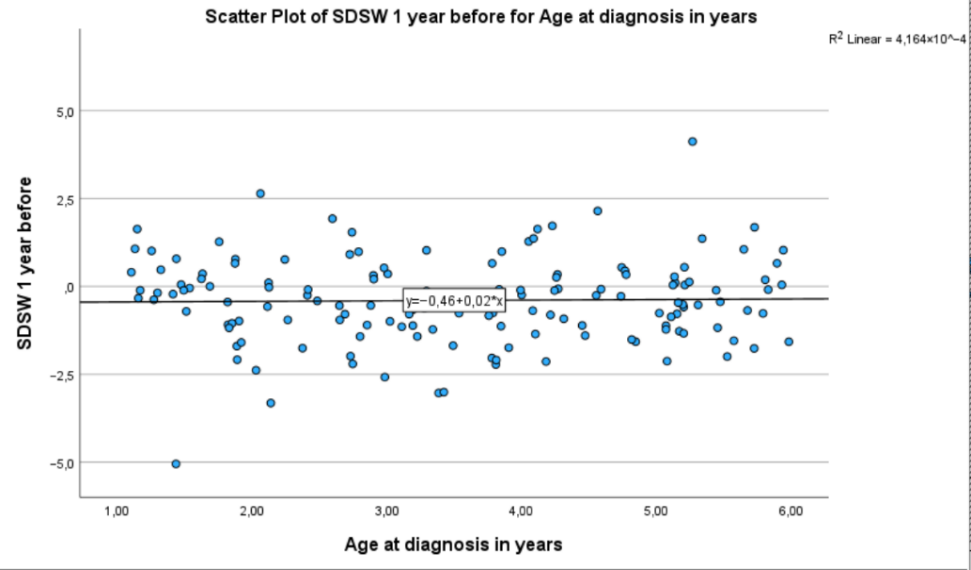


**SUPPLEMENTAL FIGURE 1 (S1):** Scatterplot of growth deviations expressed as Weight-SDS/SDSW 1 year before diagnosis at different ages where participants had their CD diagnosis.
