# Supplementary material for: Growth patterns in coeliac disease – a longitudinal study of children aged 0–6 years in Sweden
Source: BMC Pediatr. 2026 May 9;26:427. doi: 10.1186/s12887-026-06903-6 (PMC13159342; doi:10.1186/s12887-026-06903-6)
Supplement: Supplementary file 2 — Supplementary Material 2. [file 12887_2026_6903_MOESM2_ESM.docx]

**Supplemental figure 2**


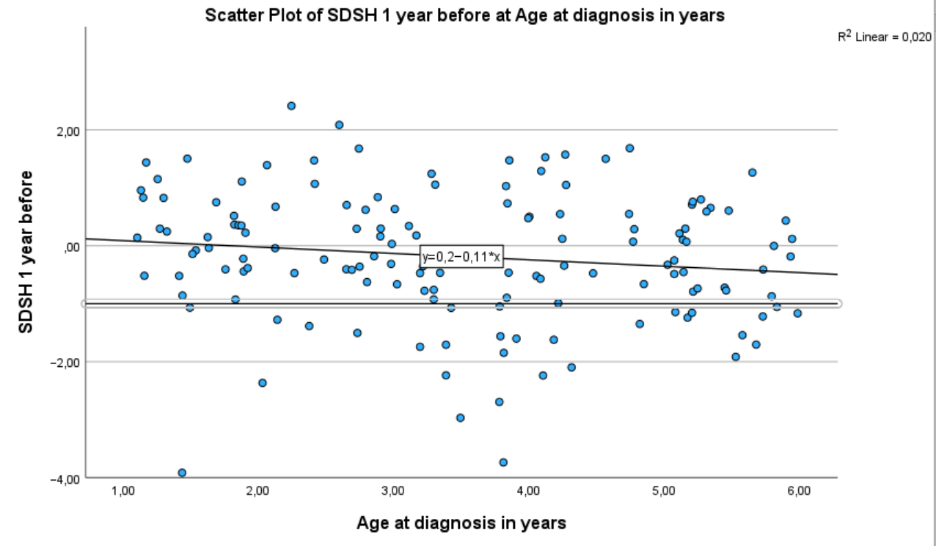


**SUPPLEMENTAL FIGURE 2 (S2):** Scatterplot of growth deviations expressed as Height-SDS/SDSH 1 year before diagnosis for different ages where participants had their CD diagnosis.
